# Associations of neighborhood walkability with moderate to vigorous physical activity: an application of compositional data analysis comparing compositional and non-compositional approaches

**DOI:** 10.1186/s12966-022-01256-6

**Published:** 2022-05-18

**Authors:** Madeleine Bird, Geetanjali D Datta, Deanna Chinerman, Lisa Kakinami, Marie-Eve Mathieu, Mélanie Henderson, Tracie A Barnett

**Affiliations:** 1 Centre de recherche du Centre hospitalier universitaire (CHU) Sainte-Justine, Montréal, Canada; 2 Département de médecine sociale et préventive, École de santé publique de l’Université de Montréal, Montréal, Canada; 3 Office of International Affairs for the Health Portfolio, Public Health Agency of Canada, Ottawa, Ontario Canada; 4 Le Centre de recherche du Centre hospitalier de l’Université de Montréal (CRCHUM), Montréal, Canada; 5 Cedars-Sinai Medical Center, Department of Medicine, Los Angeles, CA USA; 6 Department of Family Medicine, Faculty of Medicine, McGill University, 5858 Côte-des-Neiges Rd, Montreal, QC H3S 1Z1 Canada; 7 Department of Mathematics and Statistics, Concordia University, Montréal, Canada; 8 School of Kinesiology and Physical Activity Sciences, University of Montréal, Montréal, Canada; 9 Department of Pediatrics, University of Montréal, Montréal, Canada

**Keywords:** Built environment, Compositional data analysis, Moderate-to-vigorous physical activity, QUALITY cohort, Sedentary behaviour, Walkability, Youth, 24-hour movement behaviour

## Abstract

**Background:**

We compared the relation between neighborhood features and moderate to vigorous physical activity (MVPA) using linear regression analysis and the more novel compositional data analysis (CoDA). Compositional data analysis allows us to take the time children allocate to different movement behaviours during a 24-hour time period into account.

**Methodology:**

Data from youth participants (*n* = 409) in the QUALITY (QUebec Adipose and Lifestyle InvesTigation in Youth) cohort were included. Time spent in MVPA, light physical activity, sedentary behavior, and sleep (“24-hour movement behaviours”) was measured using accelerometers. Neighborhood data were collected using a geographic information system and through direct observation. In CoDA models, we used orthogonal logratio coordinates, which allows for the association of neighbourhood walkability with MVPA to be estimated with respect to the average composition of all other behaviours within a 24-hour time frame. In baseline linear regression models, MVPA was regressed cross-sectionally on neighborhood walkability. All models were stratified by sex, and controlled for BMI z-scores, pubertal development, seasonal variation, parental education, and neighbourhood safety.

**Results:**

Based on CoDA, girls who lived in more walkable neighborhoods had 10% higher daily MVPA (95% CI: 2%, 19%), taking into account all other movement behaviours. Based on linear regression, girls who resided in more walkable neighborhoods engaged in 4.2 (95% confidence interval [CI]: 1.2, 6.6) more minutes of MVPA per day on average than girls residing in less walkable neighborhoods.

**Conclusions:**

Unlike with traditional linear models, all movement behaviours were included in a single model using CoDA, allowing for a more complete picture of the strength and direction of the association between neighbourhood *Walkability* and MVPA. Application of CoDA to investigate determinants of physical activity provides additional insight into potential mechanisms and the ways in which people allocate their time.

**Supplementary Information:**

The online version contains supplementary material available at 10.1186/s12966-022-01256-6.

## Introduction

Only 9% of Canadian children aged 5–17 years meet guidelines of 60 min of moderate-to-vigorous physical activity (MVPA) per day [1]. Engaging in physical activity (PA) is essential to healthy development [2] and reduces risk of obesity in children [3].

Associations between MVPA and health outcomes are typically tested without accounting for time spent in competing behaviours [4–6]. Although there have been efforts to examine combinations of behaviours concomitantly [7], complementary movement behaviours are usually included as separate variables in a regression model [8, 9]. This more traditional approach has been criticized [10–12] for ignoring the co-dependency of movement behaviours over a 24-hour period [10, 13]. Recently, Pedisic addressed this limitation using the Activity Balance conceptual model [10]. He proposed applying a novel statistical method, compositional regression (CoDA), which accounts for the co-dependent nature of these behaviours. In contrast to traditional regression methods, CoDA would enable the calculation of the relative contribution of each behaviour to a health outcome while also accounting for the 24-hour constraint for all behaviours combined.

Studies applying CoDA to 24-hour movement behaviour have primarily described the association between the proportion of time spent in different movement behaviours and health related outcomes [11–13]. For example, over a fixed time period, less time spent in SB allows for more opportunity to engage in MVPA [13].

Neighborhoods have been theorised to influence health outcomes through their institutions and resources, via stresses in the physical and social environment, social capital, and social norms [14]. “Walkable” features of neighborhood built environments have been of particular interest as potential correlates of BMI and PA levels in children and youth [15–19], as walking has been shown to increase children’s daily MVPA levels [20, 21]. Features such as intersection density [15–17], residential density [16, 18, 19], average block length [15], mixed land use [15–18], road speeds [15, 17], traffic density [17], sidewalk coverage [15, 17, 18], crime and safety [18], and access to parks and recreation centres [17, 18] are often incorporated into a composite walkability score or index [22].

Studies of relations between walkable neighborhood features and PA among children and youth have produced mixed findings. Several systematic reviews exist on the subject, one concluding that walkability was one of the most supportive correlates of neighborhood features and children’s PA [23] while another quantified this to have relatively trivial effects on MVPA [19]. Specifically, better neighborhood walkability and walking amenities were associated with increased daily MVPA of 8 min ± 10% and 15 min ± 30% for children and adolescents, respectively [24]. Others have reported that greater walkability is associated with a decrease in physical activity [15, 25]. However, these studies used self-reported PA which may have poorly measured the outcome of interest in the youth population under study. Inconsistent findings may be due to a failure to account for key confounders and moderators including neighborhood socioeconomic status (SES) [16], age and sex, [24] in addition to inadequately accounting for the compositional properties of movement behaviours [12].

**Fig. 1:**
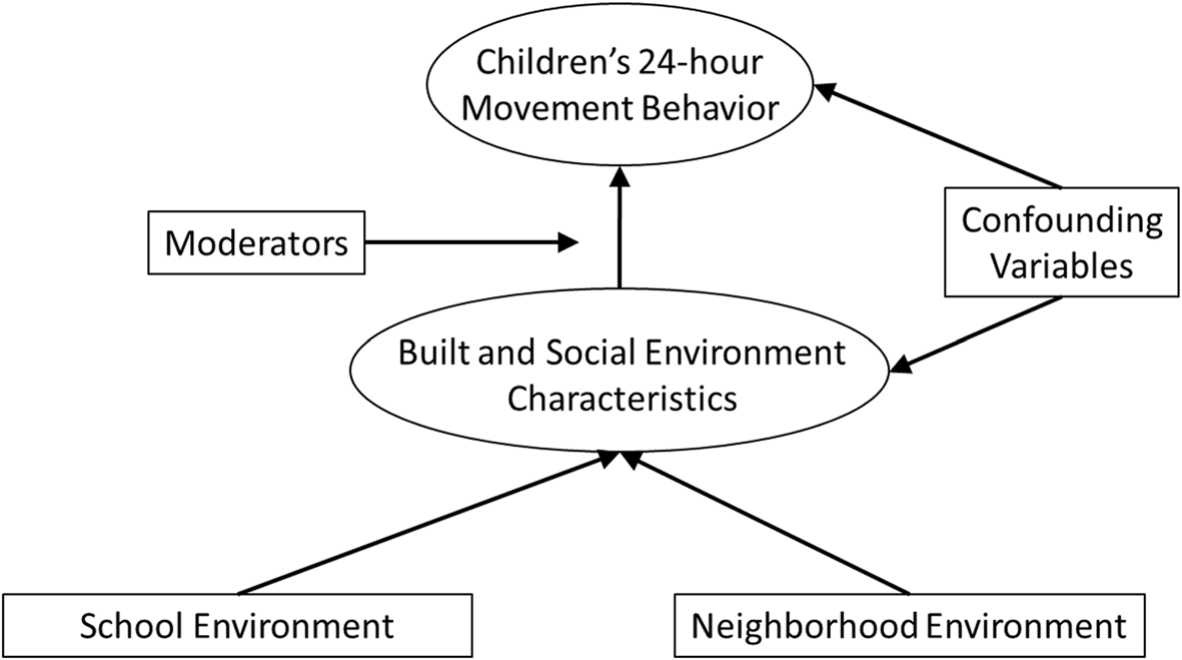
A conceptual model of the influence of the built and social environment on children’s 24-hour movement behaviour

The objective of this study was to estimate associations between neighborhood walkability and children’s MVPA among children at high risk of obesity using both CoDA, and non-compositional linear regression. This work builds on Pedisic’s Activity Balance model, and a conceptual model of how the built and social environment influences children’s 24-hour movement behaviours proposed here (Fig. 1). Although we explore the relation between neighbourhood walkability and MVPA, we do so for illustrative purposes, with the focus primarily on the comparison between conclusions derived from the two analytic methods in order to generate insights.

## Methods

### Participants

Data are from the QUebec Adipose and Lifestyle InvesTigation in Youth (QUALITY) cohort [26], an ongoing longitudinal investigation of the natural history of obesity and cardiovascular risk in Quebec youth. Participants aged 8 to 10 years were recruited through schools. A detailed description of the study design and methods is available elsewhere [26]. In brief, at least one biological parent was required to be obese for study inclusion based on parent-reported measurements of weight, height, and waist circumference (i.e., body mass index [BMI] ≥ 30 kg/m^2^ and/or waist circumference > 102 cm in men and > 88 cm in women). Among those eligible, 630 families completed baseline data collection during a research clinic visit between September 2005 and December 2008. Data collection included questionnaires completed by the child and both biological parents, and biological and physiological measurements taken from the child. Written informed consent was obtained from the parents, and assent was provided by the children. This analysis was restricted to the participants residing in the Montreal Metropolitan Area (*n* = 512) for which characteristics of the neighborhood environments were assessed.

### Exposures

Two methods were used to measure potential neighborhood walkability features: administrative data available from MEGAPHONE, a Montreal-based geographic information system (GIS), and data collected using in-person neighborhood assessments. At baseline, the exact address of each participating child’s residence was geocoded. GIS indicators were computed for 1 km street network buffers centered on the participants’ residence (ego-centered areas) [27] via CanMap (DMTI Spatial Inc., Richmond Hill, Ontario, Canada) and 2006 Canadian Census. In-person neighborhood observations were conducted by pairs of trained observers using an observation checklist adapted from existing assessment tools [28, 29] for a Canadian pediatric population. A total of 43 built environment features for each of 10 street segments (mean: 8.7) located within the immediate residential environment underwent a detailed assessment.

Using principal component analysis, a one factor solution was sought, based on the scree test. This single component, which was labelled *Walkability*, accounted for 70% in the data’s variability and included the following neighborhood indicators: (1) the number of three- or more- way intersections; (2) land use mix (residential, commercial, industrial, recreational, or other) [30]; (3) the number of parks; (4) the total length of streets with normal vehicular traffic at rush hour; (5) the proportion of the buffer area covered by parks, and (6) the number of segments with at least one sign of social disorder (such as graffiti, vandalism, litter, abandoned buildings/construction), which was captured using in-person audits. Neighborhoods with higher *Walkability* generally had more intersections (rho = 0.82), greater mixed land use (rho = 0.71), more park area ratio (rho = 0.69), higher frequency of parks (rho = 0.79), more streets with low vehicular traffic (rho = 0.86), and more signs of social disorder (rho = 0.56) than neighborhoods low on *Walkability*. Given that *Walkability* was calculated as a standardised principal component, its arithmetic mean was 0 and its standard deviation was 1. Scores were standardized and are described in greater detail in the Supplement [22].

### Outcomes

MVPA was measured using an accelerometer (Actigraph model 7184, Pensacola, Florida, USA) that was checked for calibration and fitted onto the child during the baseline visit and instructed to be worn at the hip for the following 7 consecutive days. Complying with established guidelines [31], only data from children with a minimum of 4 days with ≥ 10-hours of wear time per day were retained. All participants had at least 1 weekend day measured to be included in the analysis, with time being weighted to reflect activity patterns in a typical 7-day week. Based on established cut-offs of counts per minute (CPM), SB, LPA, and MVPA were defined as: “< 100 CPM (SB); 100–2295 CPM (LPA); and ≥ 2296 CPM (MVPA) [32]. The amount of time spent in each behaviour was averaged over a 7-day period. Mean sleep time was computed, based on the algorithm and with full-day data scans from the time when the accelerometer was removed at night until it was re-fitted in the morning. The four components of the 24-hour movement behaviour (sleep, SB, LPA, and MVPA) were standardized to a 24-hour day. This was done to correct for some non-wear time. Non-wear time was defined as periods of 60-minutes or more of “0” values were obtained, with 1–2 accepted allowance periods (1–2 consecutive minutes < 100) and was considered in calculating 24-hour movement behaviours.

### Covariates

Covariates were chosen based on existing literature [8, 14, 20] and there were no missing values for covariates in this dataset. Child anthropometrics were measured using standardized protocols [26]. Children and parents were dressed in light indoor clothing, and a calibrated stadiometer and electronic scale were used to measure height and weight respectively. World Health Organization age-and sex-specific BMI z-scores were computed [33]. Children were categorized as overweight and obese if their BMI z-score was ≥ 1 standard deviation from the mean [33]. Pubertal development stage was assessed by a nurse using the 5-stage Tanner scales [34, 35] and was dichotomized as pre-pubertal (Tanner 1) vs. puberty initiated (Tanner > 1).

Potential seasonal variation in PA was considered as a confounding variable using the month in which the accelerometer was fitted. The season variable was dichotomized as accelerometer worn between the months of May and October inclusively (i.e., summer), versus not.

The highest level of completed education achieved by both parents was reported by the child’s parents at baseline. Parental education was categorized as (1) both parents completing secondary education or less, (2) at least one parent with a technical/vocational degree but neither with completed university degree, or (3) at least one parent with a completed university degree.

Child’s perception of neighborhood safety was self-reported at baseline by responding to the question: “There is no danger when I walk or bike around my neighborhood alone during the day”. Response options were provided on a Likert-type scale where 1 = true; 2 = more or less true; 3 = more or less false: and; 4 = false. Responses were dichotomized (1 or 2 = 0; 3 or 4 = 1).

### Statistical analyses

In order to examine the association between the *Walkability* principal component and the participant’s MVPA, orthogonal logratio coordinates were used [36] with data collected at baseline (2005–2008, *n* = 409). A linear regression model for MVPA was also used to compare results between the compositional methodology and the linear regression approach. In all cases, child’s age, child’s BMI z-score, season, pubertal status, and parental education were included as covariates in the models. Analyses were restricted to participants with valid accelerometer and complete data at baseline (*n* = 409).

Results were stratified by sex to address gender-related differences in MVPA as described in the literature [37, 38]. Residual plots were examined for all models and residuals were normally distributed. Compositional means were calculated as opposed to arithmetic. Analyses were conducted using R version 3.4.1 (R Foundation for Statistical Computing, Vienna, Austria) and packages combinat [39], compositions [40], cramer [41], energy [42], gtools [43], and robustbase [44].

## Results

Baseline participant characteristics were compared between QUALITY cohort members included in and excluded from analyses (Table 1). There were no significant differences between the groups except for season with excluded participants more likely to have participated during summer months due to incomplete accelerometry data. Summer participants were more likely to be missing data, potentially due to water-based activities requiring removal of accelerometer.

**Table 1:** Baseline (2005–2008) participant characteristics and differences between included and excluded participants

Participant Characteristics	Included (*n* = 409)	Excluded (*n* = 103)	*P*-value
Age, years, mean (SD)	9.6 (0.9)	9.6 (0.9)	0.54
Female sex, % (n)	46 (190)	42 (43)	0.39
PA measured in summer, % (n)	46 (190)	60 (62)	0.01
BMI z-score, mean (SD)	1.0 (1.3)	1.2 (1.4)	0.23
Puberty initiated, % (n)	23 (95)	26 (27)	0.49
Walkability principal component, mean (SD)	0.0 (1.0)	0.1 (1.0)	0.59
At least 1 parent with a university degree, % (n)	53 (217)	53 (54)	0.94
Hours of sleep, mean (SD)^a^	10 (0.8)	10 (0.9)	0.62
Hours of sedentary time, mean (SD)^a^	6 (1.1)	6 (1.3)	0.21
Hours of light physical activity, mean (SD)^a^	7 (0.9)	7 (1.1)	0.26
Hours of moderate to vigorous physical activity, mean (SD)^a^	0.6 (0.3)	0.7 (0.3)	0.29

*SD* standard deviation, *n* number; a: data available for only 36 excluded participants

Results are presented for sex-specific MVPA using both the compositional orthogonal and linear approaches (Tables 2, 3, 4 and 5). Among girls, an increase of one unit in neighborhood Walkability (Table 2) was associated with a 10% increase in the proportion of the 24-hour period devoted to MVPA (10%; 95% CI: 2%, 19%).

**Table 2:** Relationship of neighbourhood walkability and safety (explanatory variables) with 24-hour movement behaviours (outcome variables) among girls (*n* = 190) aged 8–10 years: results of a compositional regression analysis with orthogonal logratio coordinates

	Sleep (%)				Sedentary Time (%)			Light PA (%)			MVPA (%)		
Coefficient	Estimate	95% CI			Estimate	95% CI		Estimate	95% CI		Estimate	95% CI	
Age	-2.05	-5.49	1.51		9.00	2.50	15.91	-3.87	-7.12	0.51	-2.56	-11.69	7.51
BMI z-score	-2.34	-4.32	0.31		-0.53	-3.98	3.05	0.24	-1.72	2.24	2.69	-2.97	8.67
Summer (vs. Winter)	-4.61	-9.56	0.60		-7.14	-15.27	1.76	-5.67	-10.38	-0.72	19.69	3.37	38.58
Puberty initiated (vs. uninitiated)	4.48	-2.46	11.91		16.89	3.86	31.56	-3.08	-9.28	3.54	-15.51	-30.08	2.09
At least 1 parent with a technical degree (vs. high school only)	-6.78	-12.99	0.13		-1.22	-12.26	11.20	2.51	-4.06	9.53	5.95	-12.36	28.07
At least 1 parent with a university degree (vs. high school only)	6.43	0.97	12.18		2.24	-6.61	11.93	-1.42	-6.28	3.70	-6.78	-19.36	7.76
Walkability PC	-2.73	-5.48	0.10		-4.14	-8.75	0.71	-2.69	-5.34	0.03	10.22	1.85	19.26

*PA* Physical activity, *CI* Confidence Interval, *BMI* Body mass index, *PC* Principal component (standardized), *MVPA*, Moderate-to-vigorous physical activity

**Table 3:** Relationship of neighbourhood walkability and safety (explanatory variables) with 24-hour movement behaviours (outcome variables) among boys (*n* = 219) aged 8–10 years: results of a compositional regression analysis with orthogonal logratio coordinates

	Sleep (%)			Sedentary Time (%)			Light PA (%)			MVPA (%)		
Coefficient	Estimate	95% CI		Estimate	95% CI		Estimate	95% CI		Estimate	95% CI	
Age	0.49	-2.65	3.72	8.84	3.13	14.86	-1.68	-4.44	1.17	-7.00	-14.18	0.77
BMI z-score	2.23	0.12	4.39	6.87	3.13	10.73	1.19	-0.70	3.11	-9.54	-14.20	-4.62
Summer (vs. Winter)	-4.74	-9.90	0.72	-6.30	-14.77	3.02	-3.09	-7.83	1.89	15.61	0.38	33.14
Puberty initiated (vs. uninitiated)	4.71	-5.33	15.82	5.81	-10.87	25.62	-13.85	-21.33	-5.67	4.77	-18.86	35.28
At least 1 parent with a technical degree (vs. high school only)	-1.96	-9.40	6.08	7.12	-6.33	22.51	-9.00	-15.42	-2.31	4.64	-14.32	27.80
At least 1 parent with a university degree (vs. high school only)	-0.63	-6.20	5.28	-3.26	-12.32	6.73	3.14	-2.09	8.64	0.86	-12.88	16.76
Walkability PC	− 0.0.43	-3.15	2.36	-0.46	-5.03	4.34	-0.93	-3.37	1.57	1.84	-5.06	9.24

*PA* Physical activity, *CI* Confidence Interval, *BMI* Body mass index, *PC* Principal component (standardized), *MVPA* Moderate-to-vigorous physical activity

**Table 4:** Relationship of neighbourhood walkability and safety (explanatory variables) with 24-hour movement behaviours (outcome variables) among girls (*n* = 190) aged 8–10 years: results of a linear model approach

	Sleep (hours)			Sedentary Time (hours)			Light PA (hours)			MVPA (hours)		
Coefficient	Estimate	95% CI		Estimate	95% CI		Estimate	95% CI		Estimate	95% CI	
(Intercept)	12.36	11.10	13.61	2.31	0.56	4.06	8.66	7.15	10.16	0.68	0.16	1.19
Age	-0.18	-0.31	-0.04	0.37	0.18	0.56	-0.19	-0.35	-0.03	-0.00	-0.06	0.05
BMI z-score	-0.09	-0.17	-0.01	0.0.	-0.08	0.13	0.06	-0.03	0.15	0.00	-0.03	0.04
Summer (vs Winter)	0.03	-0.17	0.23	-0.11	-0.39	0.16	-0.04	-0.28	0.20	0.12	0.04	0.20
Puberty initiated (vs uninitiated)	0.01	-0.25	0.27	0.52	0.16	0.88	-0.40	-0.70	-0.08	-0.14	-0.24	-0.03
At least 1 parent with a technical degree (vs high school only)	-0.33	-0.57	-0.07	0.07	-0.30	0.43	0.23	-0.08	0.54	0.03	-0.07	0.14
At least 1 parent with a university degree (vs high school only)	0.26	0.06	0.46	-0.02	-0.30	0.26	-0.21	-0.45	0.02	-0.03	-0.12	0.05
Walkability PC	0.01	-0.11	0.10	-0.07	-0.22	0.08	0.00	-0.13	0.13	0.07	0.02	0.11

*PA* Physical activity, *CI *Confidence Interval, *BMI *Body mass index, *PC* Principal component

**Table 5:** Relationship of neighbourhood walkability and safety (explanatory variables) with 24-hour movement behaviours (outcome variables) among boys (*n* = 219) aged 8–10 years: results of a linear model approach

	Sleep (hours)				Sedentary Time (hours)			Light PA (hours)				MVPA (hours)		
Coefficient	Estimate	95% CI			Estimate	95% CI		Estimate	95% CI			Estimate	95% CI	
(Intercept)	11.19	9.95	12.43		2.80	1.03	4.50	8.43	7.04	9.82		1.62	0.96	2.28
Age	-0.08	-0.21	0.04		0.31	0.13	0.50	-0.16	-0.30	-0.02		-0.06	-0.13	0.00
BMI z-score	-0.03	-0.12	0.05		0.18	0.07	0.30	-0.07	-0.16	0.03		-0.08	-0.13	-0.04
Summer (vs. Winter)	-0.07	-0.29	0.16		-0.10	-0.41	0.22	0.04	-0.22	0.29		0.13	0.01	0.25
Puberty initiated (vs. uninitiated)	0.38	-0.03	0.78		0.28	-0.28	0.85	-0.68	-1.14	-0.23		0.02	-0.19	0.24
At least 1 parent with a technical degree (vs. high school only)	-0.02	-0.34	0.30		0.39	-0.06	0.83	-0.39	-0.74	-0.03		0.02	-0.15	0.19
At least 1 parent with a university degree (vs. high school only)	-0.02	-0.25	0.21		-0.15	-0.47	0.17	0.17	-0.09	0.43		0.00	-0.12	0.12
Walkability PC	0.01	-0.10	0.12		-0.01	-0.16	0.15	-0.01	-0.14	0.11		0.01	-0.05	0.07

*PA* Physical activity, *CI* Confidence Interval, *BMI* Body mass index, *PC* Principal component

Using the linear approach (Table 4), a one unit increase in the *Walkability* principal component was associated with 4.2 (95% CI: 1.2, 6.6) more minutes of girls’ daily MVPA. No meaningful associations were observed for boys using either approach.

## Discussion

The aim of this study was to compare a compositional approach with a more traditional linear regression approach to examine associations between neighbourhood *Walkability* and MVPA. Compositional regression analysis showed that 8-10-year-old girls at high risk of obesity may be more likely to replace other movement behaviours with MVPA when they reside in more walkable environments. In contrast, as the linear regression approach is not designed to assess the relative dominance of one movement behaviour in relation to the others, analyses were limited to single behaviours in separate models. Although boys engaged in more MVPA per day than the girls, no association was observed between neighborhood features and boys’ MVPA. While both CoDA and linear regression identified associations between MVPA and neighbourhood walkability, it is with the CoDA approach which we can fully observe how neighbourhood walkability influences MVPA as a proportion of the 24-hour day, which behaviours MVPA displaces, and how those changes in behavioural composition impact health outcomes [45]. In the case of this study, the 10% increase in MVPA found in 8-10-year-old girls can be examined in the context of the 3%, 4%, and 3% decrease in sleep, sedentary behaviour, and LPA respectively (Table 2) resulting from an increase in neighbourhood *Walkability*. As they are part of the same model analyzing how the 24-hour day is partitioned according to movement behaviours, it can be determined that the increase in MVPA is coming from a relatively even decrease in times spent in other movement behaviours. Meanwhile, the linear models examine MVPA separately from other behaviours, and while the association can still, to some degree, be measured, the exact proportion of movement behaviours cannot. Measuring the change in proportions of movement behaviours is especially relevant for the development of interventions promoting an increase in MVPA as it allows for a more targeted approach in examining changes in behavioural patterns.

To our knowledge, this is the first study to examine associations between neighborhood walkability and MVPA among youth at high risk of obesity using a compositional approach. Although even a moderate increase in PA can garner health benefits among the least active, it is MVPA that is most important for health benefits among children [46]. Using a compositional approach, Talarico and Janssen demonstrated that relative to sleep, SB and MVPA, LPA were associated with increased BMI, waist circumference and fat mass index, while MVPA relative to the other behaviours was negatively associated with the obesity measures among children aged 10–13 years living in Kingston, Canada [12]. Considering results from the present study, neighborhood walkability features may have important obesity-related health benefits among girls at risk of obesity at the population level. Application of compositional data analysis in built environment and PA studies may help to better understand how features of the built environment influence the ways in which people spend their time over a 24-hour period. While linear regression models do not take into account compositional properties of movement behaviour data, CoDA models allow for the interpretation of movement behaviours as proportions of a whole. Analyzing movement behaviours using a compositional approach leads to not only a more nuanced understanding of how time is used and these behaviours interact, but also to a significant paradigm shift in how MVPA and other movement behaviours are understood and interpreted [20]. Regardless of behaviours or interventions, time will always remain a finite resource. How much of the 24-hour day is spent on MVPA, will always be in proportion with other behaviours which needs to be factored into policy decisions and interventions in order to design programs and environments that promote an ideal balance of movement behaviours over the course of the day.

Beyond the obvious, CoDA has the potential to be a valuable analysis technique for intervention studies, especially those focused on increasing MVPA. With interventions seeking to increase the portion of the time budget [35] spent in MVPA, CoDA analyses would provide the clearest picture of how an intervention changed how an individual or group spent their time, especially compared with linear models. Time taken out of sedentary behaviour and put towards MVPA would have different health implications than time taken out of sleep or LPA to be put towards MVPA [13]. While MVPA is the movement behaviour with the strongest association to obesity [12], it does not exist in a vacuum and the purpose of an MVPA intervention would not only be to increase MVPA but to also promote the optimal combination of movement behaviours throughout the 24-hour day. CoDA may be the best method to use here because, in one model, it demonstrates the entire balance of not just MVPA but all movement behaviours, making changes in said behaviours easier to understand and interpret.

Intensity zones (sleep, SP, LPA, MVPA) represent only one dimension of 24-hour movement behaviour [47]. Other dimensions include posture/activity type (reclining, sitting, standing, walking, running, cycling, and walking stairs), bout duration (short, moderate, or long), domain (sleep, work/school, and non-work), and biological state (awake or asleep) [40]. Further studies analysing the relationship between the built environment and 24-hour movement behaviours would benefit from studying multiple dimensions of 24-hour movement behaviours in order to provide a more thorough and complex understanding of how individuals move through their environment throughout the day.

This study’s strengths include well defined and objectively measured neighborhood features as well as accelerometer-derived movement behaviours. Using the compositional approach based on orthogonal logratio coordinates was another strength and a novel aspect of this study. This is a novel methodology in examining the association between neighborhood walkability features, as well as other built environment features and 24-hour movement behaviours.

This study had several limitations. Sleep time was estimated as the time the accelerometer was removed at night to the time it was replaced in the morning and thus some non-wear time while awake might have been misclassified as sleep. Participants with valid accelerometer data were less likely to have had their PA measured during summer months than those who were not included, which may have biased results toward the null as participants may have been less physically active in their neighborhood during non-summer months. Participants were not fitted with global positioning systems, so data were not available on where they were engaging in their PA. Finally, this is a data-driven exploratory study which is useful to help in guiding future research. Although this study did not include a representative sample of children, results may be generalizable to children who are overweight, obese, or at high risk of being so. As currently almost 30% of Canadian children 5 to 17 years are overweight or obese [48], this represents a significant proportion of the pediatric population.

## Conclusions

Both the compositional approach and linear models demonstrate an association between an increase in MVPA relative to walkable neighbourhood features, but through CoDA it is also possible to observe the decrease in sleep, SB, and LPA relative to MVPA. Using the compositional data approach provided novel insight into the association between built environment features and the relative time spent in different activities over a 24-hour period and may be applicable to a wide range of studies examining the association between built environment features, and other determinants of behaviour, and 24-hour movement behaviours. Future research should confirm the findings in a larger sex-stratified sample, analyze the effect of how parental perceptions of environmental safety impact 24-hour movement behaviour in children, and replicate the approach for other health behaviours. Exploring other features of the built environment and 24-hour movement behaviours to gain a better insight into how the built environment impacts on the relative time children spend in each activity over a day is warranted.

## Supplementary Information

**Additional file 1.**

## Data Availability

Data are available from the corresponding author on reasonable request.

## Abbreviations

BMI: Body mass index
MVPA: Moderate-to-vigorous physical activity
QUALITY: QUebec Adipose and Lifestyle InvesTigation in Youth
LPA: Light physical activity
SB: Sedentary behaviour
CoDA: Compositional data analysis
PA: Physical activity
CHU: Centre Hospitalier Universitaire
GIS: Geographic information system
CPM: Counts per minute
CI: Confidence Interval

## References

[1] ParticipACTION (2016). Are Canadian kids too tired to move? The 2016 ParticipACTION Report Card on Physical Activity for Children and Youth.

[2] Centers for Disease Control and Prevention. Increasing physical activity: a report on recommendations of the Task Force on Community Preventive Services. 2001. Contract No.: No. RR-18. 11699650

[3] Trost SG, Kerr LM, Ward DS, Pate RR (2001). Physical activity and determinants of physical activity in obese and non-obese children. Int J Obes Relat metabolic disorders: J Int Association Study Obes.

[4] Maher C, Olds T, Mire E, Katzmarzyk PT (2014). Reconsidering the Sedentary Behaviour Paradigm. PLoS ONE.

[5] Henson J, Yates T, Biddle SJ, Edwardson CL, Khunti K, Wilmot EG (2013). Associations of objectively measured sedentary behaviour and physical activity with markers of cardiometabolic health. Diabetologia.

[6] Santos R, Mota J, Okely AD, Pratt M, Moreira C, Coelho-e-Silva MJ (2014). The independent associations of sedentary behaviour and physical activity on cardiorespiratory fitness. Br J Sports Med.

[7] Saunders TJ, Gray CE, Poitras VJ, Chaput J-P, Janssen I, Katzmarzyk PT (2016). Combinations of physical activity, sedentary behaviour and sleep: relationships with health indicators in school-aged children and youth. Appl Physiol Nutr Metab.

[8] Wilkie HJ, Standage M, Gillison FB, Cumming SP, Katzmarzyk PT (2016). Multiple lifestyle behaviours and overweight and obesity among children aged 9–11 years: results from the UK site of the International Study of Childhood Obesity, Lifestyle and the Environment. BMJ Open.

[9] Katzmarzyk PT, Barreira TV, Broyles ST, Champagne CM, Chaput J-P, Fogelholm M (2015). Relationship between lifestyle behaviors and obesity in children ages 9–11: Results from a 12-country study. Obesity.

[10] Pedisic Z (2014). Measurement issues and poor adjustments for physical activity and sleep undermine sedentary behaviour research -- the focus should shift to the balance between sleep, sedentary behaviour, standing and activity. Kinesiology.

[11] Carson V, Tremblay MS, Chaput J-P, Chastin SFM (2016). Associations between sleep duration, sedentary time, physical activity, and health indicators among Canadian children and youth using compositional analyses. Appl Physiol Nutr Metab.

[12] Talarico R, Janssen I. Compositional associations of time spent in sleep, sedentary behavior and physical activity with obesity measures in children. Int J Obes. 2018. 10.1038/s41366-018-0053-x 29568110

[13] Chastin SFM, Palarea-Albaladejo J, Dontje ML, Skelton DA (2015). Combined Effects of Time Spent in Physical Activity, Sedentary Behaviors and Sleep on Obesity and Cardio-Metabolic Health Markers: A Novel Compositional Data Analysis Approach. PLoS ONE.

[14] Ellen IG, Mijanovich T, Dillman K-N (2001). Neighborhood Effects on Health: Exploring the Links and Assessing the Evidence. J Urban Affairs.

[15] Laxer RE, Janssen I (2013). The proportion of youths’ physical inactivity attributable to neighbourhood built environment features. Int J Health Geogr.

[16] D’Haese S, Van Dyck D, De Bourdeaudhuij I, Deforche B, Cardon G (2014). The association between objective walkability, neighborhood socio-economic status, and physical activity in Belgian children. Int J Behav Nutr Phys Act.

[17] Duncan DT, Sharifi M, Melly SJ, Marshall R, Sequist TD, Rifas-Shiman SL (2014). Characteristics of walkable built environments and BMI z-scores in children: evidence from a large electronic health record database. Environ Health Perspect.

[18] De Meester F, Van Dyck D, De Bourdeaudhuij I, Cardon G (2014). Parental perceived neighborhood attributes: associations with active transport and physical activity among 10–12 year old children and the mediating role of independent mobility. BMC Public Health.

[19] Buchan DS, Ollis S, Thomas NE, Baker JS. Physical Activity Behaviour: An overview of current and emergent theoretical practices. J Obes. 2012. 10.1155/2012/546459 PMC3388376 22778918

[20] Davison KK, Werder JL, Lawson CT (2008). Children’s active commuting to school: current knowledge and future directions. Prev Chronic Dis.

[21] Lee MC, Orenstein MR, Richardson MJ (2008). Systematic Review of Active Commuting to School and Children’s Physical Activity and Weight. J Phys Activity Health.

[22] Tracie A, Barnett AEG, Van Hulst A, Contreras G, Kestens Y, Chaix B. Marie-Soleil Cloutier, Melanie Henderson. Neighborhood built environment typologies and adiposity in children and adolescents. Int J Obes. 10.1038/s41366-021-01010-1. 2021. [Online ahead of print]. 10.1038/s41366-021-01010-1 34848835

[23] Ding D, Sallis JF, Kerr J, Lee S, Rosenberg DE (2011). Neighborhood Environment and Physical Activity Among Youth: A Review. Am J Prev Med.

[24] McGrath LJ, Hopkins WG, Hinckson EA (2015). Associations of Objectively Measured Built-Environment Attributes with Youth Moderate–Vigorous Physical Activity: A Systematic Review and Meta-Analysis. Sports Med.

[25] Janssen I, King N (2015). Walkable school neighborhoods are not playable neighborhoods. Health Place.

[26] Lambert M, van Hulst A, O’Loughlin J, Tremblay A, Barnett T, Charron H, et al. Cohort Profile: The Quebec Adipose and Lifestyle Investigation in Youth Cohort. Int J Epidemiol. 2012:1–12. 10.1093/ije/dyr111 21785124

[27] Chaix B (2009). Geographic Life Environments and Coronary Heart Disease: A Literature Review, Theoretical Contributions, Methodological Updates, and a Research Agenda. Annu Rev Public Health.

[28] Pikora T, Bull F, Jamrozik K, Knuiman M, Giles-Corti B, Donovan R (2002). Developing a Reliable Audit Instrument to Measure the Physical Environment for Physical Activity. Am J Prev Med.

[29] Paquet C, Cargo M, Kestens Y, Daniel M (2010). Reliability of an instrument for direct observation of urban neighbourhoods. Landsc Urban Plann.

[30] Leslie E, Butterworth I, Edwards M. Measuring the walkability of local communities using Geographic Information Systems data. Walk21-VII, “The Next Steps”, The 7th International Conference on Walking and Liveable Communities; October 23–25; Melbourne, Australia2006.

[31] Colley R, Garriguet D, Janssen I, Craig CL, Clarke J, Tremblay MS (2011). Physical activity of Canadian children and youth: Accelerometer results from the 2007 to 2009 Canadian Health Measures Survey. Health Rep.

[32] Evenson KR, Catellier DJ, Gill K, Ondrak KS, McMurray RG (2008). Calibration of two objective measures of physical activity for children. J Sports Sci.

[33] de Onis M, Onyango A, Borghi E, Siyam A, Nishida C, Siekmann J (2007). Development of a WHO growth reference for school-aged children and adolescents. Bull World Health Organ.

[34] Marshall W, Tanner J (1969). Variations in Pattern of Pubertal Changes in Girls. Arch Dis Childh.

[35] Marshall W, Tanner J (1970). Variations in the Pattern of Pubertal Changes in Boys. Arch Dis Child.

[36] Muller I, Hron K, Fiserova E, Smahaj J, Cakirpaloglu P, Vancakova J (2018). Interpretation of Compositional Regression with Application to Time Budget Analysis. Austrian J Stat.

[37] Carver A, Salmon J, Campbell K, Baur L, Garnett S, Crawford D (2005). How do perceptions of local neighborhood relate to adolescents’ walking and cycling?. Am J Health Promotion.

[38] van Loon J, Frank LD, Nettlefold L, Naylor P-J (2014). Youth physical activity and the neighbourhood enivronment: Examining correlates and the role of neighbourhood definition. Soc Sci Med.

[39] Chasalow S. combinat: combinatorics utilities. R package version 0.0–8. 2012.

[40] Gerald K, van den Boogaart RT-D, Matevz Bren,. compositions: Compositional Data Analysis. R package version 2.0–2. 2021.

[41] Franz C cramer: Multivariate Nonparametric Cramer-Test for the Two-Sample-Problem. R package version 0.9-3. 2019.

[42] Maria Rizzo GS. energy: E-Statistics: Multivariate Inference via the Energy of Data. R package version 1.7-8. 2021.

[43] Gregory R, Warnes BB. Thomas Lumley. gtools: Various R Programming Tools. R package version 3.9.2. 2021.

[44] Martin Maechler PR, Christophe Croux V, Todorov A, Ruckstuhl M, Salibian-Barrera T, Verbeke, Manuel Koller, Eduardo Conceicao, Maria Anna di Palma. robustbase: Basic Robust Statistics. R package version 0.93-9. 2021.

[45] McGregor DE, Palarea-Albaladejo J, Dall PM, Del Pozo Cruz B, Chastin SF. Compositional analysis of the association between mortality and 24-hour movement behaviour from NHANES. Eur J Prev Cardiol. 2019:2047487319867783. 10.1177/2047487319867783 34247228

[46] Janssen I, LeBlanc A (2010). Systematic review of the health benefits of physical activity and fitness in school-aged children and youth. Int J Behav Nutr Phys Activity.

[47] Stevens ML, Gupta N, Inan Eroglu E, Crowley PJ, Eroglu B, Bauman A (2020). Thigh-worn accelerometry for measuring movement and posture across the 24-hour cycle: a scoping review and expert statement. BMJ Open Sport Exerc Med.

[48] Statistics Canada (2015). Overweight and obesity in children and adolescents: Results from the 2009 to 2011 Canadian Health Measures Survey.

