# Supplementary material for: Associations of neighborhood walkability with moderate to vigorous physical activity: an application of compositional data analysis comparing compositional and non-compositional approaches
Source: Int J Behav Nutr Phys Act. 2022 May 18;19:55. doi: 10.1186/s12966-022-01256-6 (PMC9118591; doi:10.1186/s12966-022-01256-6)
Supplement: Supplementary file 1 — Additional file 1. [file 12966_2022_1256_MOESM1_ESM.doc]

STROBE Statement—Checklist of items that should be included in reports of ***cohort studies***

|  | Item No | Recommendation |
| --- | --- | --- |
| **Title and abstract** | 1 | (*a*) Indicate the study’s design with a commonly used term in the title or the abstract Page 3 |
|  |  | (*b*) Provide in the abstract an informative and balanced summary of what was done and what was found  Pages 3-4, lines 48-74 |
| Introduction | | |
| Background/rationale | 2 | Explain the scientific background and rationale for the investigation being reported  Pages 4-6 |
| Objectives | 3 | State specific objectives, including any prespecified hypotheses  Page 6, lines 117-124 |
| Methods | | |
| Study design | 4 | Present key elements of study design early in the paper  Page 6, lines 127-130. |
| Setting | 5 | Describe the setting, locations, and relevant dates, including periods of recruitment, exposure, follow-up, and data collection  Pages 6-7, lines 133-139 |
| Participants | 6 | (*a*) Give the eligibility criteria, and the sources and methods of selection of participants. Describe methods of follow-up  Page 6, lines 129-133 |
|  |  | (*b*) For matched studies, give matching criteria and number of exposed and unexposed  N/A |
| Variables | 7 | Clearly define all outcomes, exposures, predictors, potential confounders, and effect modifiers. Give diagnostic criteria, if applicable  Pages 7-9, subsections “Exposure”, “Outcomes”, and “Covariates” |
| Data sources/ measurement | 8* | For each variable of interest, give sources of data and details of methods of assessment (measurement). Describe comparability of assessment methods if there is more than one group  Pages 7-9, subsections “Exposure”, “Outcomes”, and “Covariates” |
| Bias | 9 | Describe any efforts to address potential sources of bias  Page 6 lines 127-130, Page 10 lines 206-215 |
| Study size | 10 | Explain how the study size was arrived at  Page 6 lines 133-135, Page 10 lines 211-212 |
| Quantitative variables | 11 | Explain how quantitative variables were handled in the analyses. If applicable, describe which groupings were chosen and why  Pages 7-9, subsections “Exposure”, “Outcomes”, and “Covariates” |
| Statistical methods | 12 | (*a*) Describe all statistical methods, including those used to control for confounding  Page 10, subsection “Statistical Analysis” |
|  |  | (*b*) Describe any methods used to examine subgroups and interactions  Page 10, lines 213-214 |
|  |  | (*c*) Explain how missing data were addressed  Page 10. Lines 211-212 |
|  |  | (*d*) If applicable, explain how loss to follow-up was addressed  Page 6, lines 127-130 |
|  |  | (*e*) Describe any sensitivity analyses  N/A |
| Results | | |
| Participants | 13* | (a) Report numbers of individuals at each stage of study—eg numbers potentially eligible, examined for eligibility, confirmed eligible, included in the study, completing follow-up, and analysed  Page 10 lines 220-221, Table 1 |
|  |  | (b) Give reasons for non-participation at each stage  Page 10 lines 221-224, Table 1 |
|  |  | (c) Consider use of a flow diagram  N/A |
| Descriptive data | 14* | (a) Give characteristics of study participants (eg demographic, clinical, social) and information on exposures and potential confounders  Page 10 lines 220-221, Table 1 |
|  |  | (b) Indicate number of participants with missing data for each variable of interest  Page 10 lines 220-221, Table 1 |
|  |  | (c) Summarise follow-up time (eg, average and total amount)  N/A |
| Outcome data | 15* | Report numbers of outcome events or summary measures over time  Supplemental Materials |
| Main results | 16 | (*a*) Give unadjusted estimates and, if applicable, confounder-adjusted estimates and their precision (eg, 95% confidence interval). Make clear which confounders were adjusted for and why they were included  Pages 10-11 lines 225-231, Tables 2-5 |
|  |  | (*b*) Report category boundaries when continuous variables were categorized  Pages 10-11 lines 225-231, Tables 2-5 |
|  |  | (*c*) If relevant, consider translating estimates of relative risk into absolute risk for a meaningful time period  N/A |
| Other analyses | 17 | Report other analyses done—eg analyses of subgroups and interactions, and sensitivity analyses  Supplemental Materials |
| Discussion | | |
| Key results | 18 | Summarise key results with reference to study objectives  Pages 11-12, lines 242-253 |
| Limitations | 19 | Discuss limitations of the study, taking into account sources of potential bias or imprecision. Discuss both direction and magnitude of any potential bias  Page 14, lines 304-315 |
| Interpretation | 20 | Give a cautious overall interpretation of results considering objectives, limitations, multiplicity of analyses, results from similar studies, and other relevant evidence  Pages 12-13, lines 259-290 |
| Generalisability | 21 | Discuss the generalisability (external validity) of the study results  Page 314, lines 312-314 |
| Other information | | |
| Funding | 22 | Give the source of funding and the role of the funders for the present study and, if applicable, for the original study on which the present article is based  Page 16, lines 342-349 |

*Give information separately for exposed and unexposed groups.

**Note:** An Explanation and Elaboration article discusses each checklist item and gives methodological background and published examples of transparent reporting. The STROBE checklist is best used in conjunction with this article (freely available on the Web sites of PLoS Medicine at http://www.plosmedicine.org/, Annals of Internal Medicine at http://www.annals.org/, and Epidemiology at http://www.epidem.com/). Information on the STROBE Initiative is available at http://www.strobe-statement.org.
